# Fracture of the Internal Metatarsal, or Internal Splint Bone

**Published:** 1883-04

**Authors:** Frank V. Walton

**Affiliations:** Surgeon to Columbia Veterinary College Hospital


### X.—FRACTURE OF THE INTERNAL METATARSAL, OR INTERNAL SPLINT BONE.

BY FRANK V. WALTON, D.V.S.,

*Surgeon to Columbia Veterinary College Hospital.*

Gray gelding, aged 12, occupation, heavy truck horse.

While pulling a heavy load on Broadway he lost his footing, slipped, and suddenly became very lame.

The animal was brought to the hospital, and upon examination the internal metatarsal bone, commonly called splint bone, was found to have been fractured very near its superior articular surface.

The horse was kept at perfect rest, and warm applications applied until the swelling disappeared. This having been accomplished a plaster of paris splint was applied and the animal partially supported by slings for a short time.

At the end of thirty days the splints were removed and complete union was found to have taken place. The animal was kept at rest for five days longer when he was permitted to attempt slow work.

[It occurs to the editors that this was an unusually long time to lay an animal up for so trivial an injury as the fracture of one of these small and accessory bones. Had it been one of the main bones of support it probably would have taken all this and more time.—ED.]

